# “A doctor who really knows …”: a survey of community perspectives on medical students and practitioners with disability

**DOI:** 10.1186/s12909-019-1715-7

**Published:** 2019-07-29

**Authors:** Lise Mogensen, Wendy Hu

**Affiliations:** Western Sydney University, School of Medicine, Ainsworth Building, Campbelltown Campus, Locked Bag 1797, Penrith, NSW 2751 Australia

**Keywords:** Widening participation, Equity, Inclusion, Disability, Medical student, Medical education

## Abstract

**Background:**

In Australia, the proportion of medical students with disability remains low compared to students with disability in other university courses and to the prevalence of disability in society. Arguments for inclusion include medical school obligations to respond to community values in their programs, and that doctors with disabilities can offer valuable insights for patient care from their experiences. This study aimed to inform inclusive and socially accountable medical programs by investigating community views on doctors and medical students with disability.

**Methods:**

A concurrent mixed methods study was conducted, simultaneously collecting quantitative fixed responses, and qualitative free text responses to provide in-depth and triangulated data on community views. Frequency and thematic analysis within and across response categories was used to identify patterns and relationships, providing context and meaning to the quantitative data for the integrated findings.

**Results:**

Of 207 respondents aged 17 to 71 years, 71% were female, and 60.2% had university level education. Most (92.3%) knew someone with a long standing disability, illness, mental health condition or learning difficulty, 74.7% agreed that a person with a disability should be encouraged to study medicine, 79.7% agreed that a person with a disability should be accepted into medical school, and 81.4% that including people with disability would be an advantage in the medical profession. Five integrated themes explained these views: 1) Fair selection, support and monitoring is expected of medical schools, 2) Life experiences of disability promotes real empathy in doctors, 3) Career considerations for those with disability, 4) Medical role models to address disabling social barriers, and 5) Responsibility to monitor own health and ability to perform.

**Conclusions:**

This study indicates Australian community support for inclusion of people with disability as medical students and practitioners. Findings also suggest community expectations and trust in medical schools to effectively select and graduate only those who will be capable doctors, and to support health and development of all students towards being competent graduates. These findings provide support for medical schools to develop inclusive practices in medical education and training relevant to the health services and communities they serve.

**Electronic supplementary material:**

The online version of this article (10.1186/s12909-019-1715-7) contains supplementary material, which is available to authorized users.

## Background

Inclusion and accommodation of medical students with disabilities has been debated in United Kingdom (UK), United States (US) and Canadian literature since the 1980’s. Arguments for inclusion include the obligation for medical schools to respond to community and public values given the place of doctors in society in their curricula, that doctors with disabilities can practice competently, and that they can offer unique insights to their patients on the basis of their own experiences [1, 2]. More recently, this debate has been fuelled again by the adoption of widening participation in education policies in many countries [3–6]. In the UK the General Medical Council is actively promoting such policies to attract, support and enable more students with disabilities to participate in medical education and to encourage a more diverse medical workforce [7]. Extensive reviews of the complex issues around studying and practicing medicine with a disability has led to revision of admission criteria and processes, and guidance on suitable adjustments to educational practice in UK medical schools, that will still meet the essential requirements of medical doctors [1, 7]. In the US there has been much emphasis on policy change [8, 9] and the specific strategies to deliver reasonable adjustments and accommodations [10]. There is little direct evidence, however, about general community views and values on the inclusion of people with disability in medical education and training, despite these efforts and initiatives.

Higher education widening participation policies were launched in Australia with funding targeted at increasing and supporting the enrolment of students with disabilities in university and post-secondary courses [11]. However, so far the main change has been to include the key tenets of anti-discrimination legislation in the standards for medical education providers [6]. There is yet to be an open exploration of how to best include students with disabilities in Australian medical education. The number of medical students with disability in Australia remains low (less than 2%) compared to other post-secondary courses (around 5%) [12], compared to UK medical schools (approximately 4.1%) [13]. As Australian research on doctors and medical students with disabilities is largely absent, the reasons for underrepresentation are speculative. Due to non-disclosure, it is difficult to accurately identify the number of students with disabilities being accepted into medical schools, with official figures not reflecting actual numbers of individuals with disabilities studying medicine [14]. UK researchers [15] attribute the main reasons for non-disclosure to factors such as fear of discrimination and presumed incompetence, and concerns about lack of understanding amongst patients, colleagues and the general community of what constitutes a disability.

Over the past 30 years, community attitudes show an increasing acceptance towards persons with disability [16], but there is little known specifically about community views on and attitudes toward doctors, health professionals or health and medical students with disabilities. It is also not known whether there is broad community support of widening participation in the medical profession with respect to disability. A qualitative study by Roberts and Boursicot [1] showed variable views among focus group participants in the UK, including six participants from the general public. Some participants suggested that ‘disabled doctors might empathise better’ with patients, while others thought that ‘disabled doctors might be limited in what they could do’ and as such that there would be questions of cost versus value in educating people with disabilities to become doctors.

We aimed to address this limited evidence base by surveying the lay community about their views on disability and on widening participation in medical education and training to be inclusive of people with a disability. With such evidence, medical schools may be better equipped to respond to policy and legislative change, and to be accountable to the community in their education programs. Our research question was: What are the views and attitudes of the lay community towards the inclusion of medical students and doctors with a disability in medical education and in the medical profession?

## Methods

### Study design

Adopting Bazeley’s [17] mixed method typology and approach to data collection, analysis and interpretation, a cross-sectional survey was conducted to collect quantitative and qualitative data for a concurrent, complementary, mixed methods study. The complementary design enabled simultaneous collection and analysis of numerical and text data that could contextualise frequency statistics with a more nuanced understanding of community views. The study was approved by the Western Sydney University Human Research Ethics committee [ID no: H9989].

### Setting and recruitment of participants

The Greater Western Sydney region in New South Wales Australia is a diverse, outer metropolitan area spanning over more than 894.000 ha. It is a multicultural community with the fastest growing population in Australia, currently near 2.3 million. In 2016, the dominant occupational groups were Professionals (20.0%), Clerical and Administrative Workers (15.8%) and Technicians and Trades Workers (13.7%) [18].

Participants from the general community were recruited to the survey by convenience sampling using a Western Sydney University social media platform and emails to community organisations, university groups, and General Practice managers for distribution to Greater Western Sydney community networks. Respondents residing in Australia aged 17 and over were eligible to participate via an online survey. Study information was provided at the beginning of the survey and consent was implied by participants completing and submitting their responses.

### Data collection

Survey questions on attitude toward disability in the medical profession were adapted from the British Social Attitude survey by Park and colleagues [19]. For example, a question in the British Attitude survey such as *“How would you feel if a person using a wheel chair was to move in next door?”* was changed to *“How would you feel if a person using a wheel chair was your doctor?”* rated on a five-point Likert scale (Very comfortable, fairly comfortable, fairly uncomfortable, very uncomfortable, not sure)*.* Open ended questions were developed for the survey, based on concepts from focus group interviews by Roberts et al. [1] and aimed to explore in more detail the underlying concepts behind community views and attitudes to doctors with disability. For this purpose, fixed answer questions were asked, such as *“Generally speaking, should a person with a disability or chronic condition be accepted into medical school?”* with the response options: yes, no or not sure. These were followed with a question seeking further explanation for their response selection in free text. Some additional questions were developed specifically for this survey to explore respondents’ experiences with disability and their views more generally on people with disability, and educational options for them. Demographic questions included age, gender, ethnicity, level of education, occupation and postcode. No personally identifying details were requested from respondents. Skip logic was applied to some answers, which guided respondents to the next relevant question and past irrelevant questions. The survey was trialled with academic and professional university staff and questions revised for clarity. [see Additional file 1: Survey Questions].

### Data analysis

Quantitative data were analysed descriptively. Qualitative data were initially coded within their respective response categories and then analysed thematically across response categories to enable exploration of patterns in, and relationships between data. LM reviewed all open ended responses and initially categorised these into themes on which the survey was based, focusing on both majority held, and countervailing views and attitudes about:

1. the idea of doctors with disability, and
2. widening participation in medical education to be more inclusive of students with disability.

Within these broad categories, the final themes were identified through several reiterations of open coding, and refining concepts by both authors. This process helped to explore data in greater depth than might otherwise be concealed by presenting individually grouped responses [17]. Following a mixed methods approach, the qualitative and quantitative analyses were treated as complementary. Frequencies of fixed responses are co-presented with conceptually related qualitative themes to provide a more coherent and nuanced picture [20] of community views on disability in medical education and practice.

## Results

The survey was accessed by 209 community members. Two responses were removed due to suggested low age. Answers were not forced, which resulted in some missing data. The findings below focus on responses that illustrate views on including people with disability in the medical profession. These questions were completed by between 168 to 191 respondents. In the descriptive statistics, the reported ‘n’ reflects the number of responses to individual questions.

### Respondent characteristics

Respondents’ ages ranged from 17 to 71 years (mean age 37.58 year; SD 15.73). The vast majority of respondents were from the state of New South Wales (94%), with over two thirds of postcodes in the Western (48%) and South Western Sydney (24%) area. Most identified as female (71%), with 11% as male, and 18% as other. Many listed multiple ethnic backgrounds, but more than half (53%) included Australian as all or part of their ethnicity. Most had graduate or post-graduate qualifications, and occupations included university students, academics, health professionals, doctors and other professionals and managers. Table 1 outlines respondents’ characteristics.

**Table 1:** Participant Characteristics

**Age group**	**Percentage**	***n***
17–25 years	34.1%	58
26–35 years	15.3%	27
36–45 years	14.7%	25
46–55 years	20%	35
56–65 years	10.6%	18
65+ years	5.3%	9
Total	100%	172
**Ethnic identity**	**Percentage**	***n***
Multiple	38%	64
Australian	53%	89
Anglo-Euro-Caucasian	35%	59
Asian	11%	18
Other	14%	23
Total	100%	168
**Highest Level Education**	**Percentage**	***n***
Primary school	0.00%	0
Secondary school	19.30%	33
Post school or trade Certificate	7.60%	13
Diploma	12.90%	22
Graduate university degree	22.80%	40
Post-graduate university degree	37.40%	64
Total	100%	172
**Occupation**	**Percentage**	***n***
Academic	16%	12
Disability Sector Professional	16%	12
Health Professional	14%	10
Other Professional or Manager	14%	10
Medical student	11%	8
University student	9%	7
Doctor	7%	5
Other	14%	10
Total	100%	74
**Postcode location**	**Percentage**	***n***
NSW	94%	162
-Western Sydney^1^	51%	82
-South Western Sydney^1^	26%	42
-Sydney Metropolitan^1^	16%	26
-NSW other^1^	7%	12
Queensland	4%	7
Victoria	2%	3
Total	100%	172

The number for this region is encompassed in total NSW numbers

The vast majority (92.3%, *n* = 191 of 207) indicated that they knew someone with a long standing physical illness, disability, mental health condition or learning difficulty, most often a relative or a close friend (see Table 2). Of these respondents, more than two thirds (68%) knew someone with mental illness (depression, 45%, schizophrenia, 16% or other, 7%), and around a quarter knew someone with learning or intellectual disability (25. 6%) or autism (24.6%), while slightly fewer knew someone using a wheelchair (20.9%), or with hearing (22.5%) or vision (14.1%) impairment.

**Table 2:** Respondents’ relationship to persons with disability or chronic condition

Persons with disability known to respondents	Percentage	*n* = 191
A relative^a^	49.7%	86
A close friend	34.7%	60
A person in your local community^a^	28.9%	50
A colleague or co-worker	16.2%	28
Your partner	14.5%	25
Other person	13.9%	24
Your child	9.3%	17
Self^b^	8.7%	15
Your health service provider	0.6%	1
Your boss	0.6%	1

Total does not add to 100% due to multiple selections

^a^Additional free text responses included in this category

^b^New category emerged from free text responses

### Integrated themes: people with disability in the medical profession

**Fig. 1:**
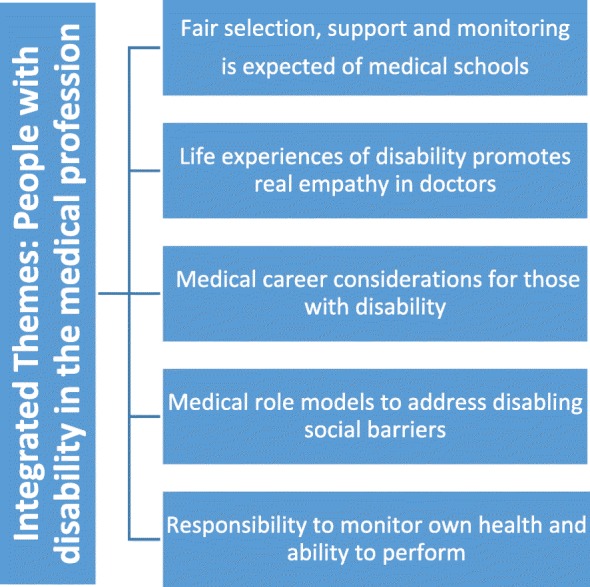
Integrated key themes from qualitative data

Most respondents (89.9%, *n* = 169 of 188)] indicated that persons with a disability should be able and encouraged to be educated or trained in a career of their choice. As the following sections show, they also held positive views towards training students with a disability to become doctors. Some respondents speculated on the potential difficulties that medical education and practice might present for people with certain impairments. However, the responsibility to assess capacity fairly and to support students with disability was placed squarely on medical schools, with respondents trusting medical schools to do this effectively. Qualitative data is presented to contextualise, further explore and explain the meanings to the majority views in the fixed responses, as well as explore the reasons for outlier and contrary views. Five thematic categories emerged from the integrated analysis as illustrated in Fig. 1 and below.

### Fair selection, support and monitoring is expected of medical schools

The vast majority (79.7%, *n* = 145 of 182) agreed that a person with a disability should be accepted into medical school, while 30 (16.5%) were unsure and seven said no. Some respondents wondered whether individuals with some conditions were more suitable than others, but most expressed trust in medical schools to employ a fair and equal selection process to select those with the ability and capacity to study medicine and not based on *‘…whether they are disabled or not’ [#22].* The need to assess the ability of all individuals to complete the medical course and practice medicine safely was also strongly emphasised by some respondents:

*‘…all persons who have capability should be allowed to pursue this [and any] career path, provided they can perform the tasks of the role’* [#64]

*“The expectations of the individual need to be considered on a case by case level to identify if medical school is going to lead to career development for the individual”* [#58]*.*

In addition to a fair selection process, an implicit expectation was expressed that medical schools should monitor the progress and capacity of individuals over time into graduation:

*“… medical school will determine if they can't continue”* [#46]

*“Most … would require careful guidance in what areas of medicine would best suit their ability or patients should not be negatively impacted by their disability”* [#203]

Some stated that there was little need for medical schools to widen participation, but that people with capacity to study and practice medicine should not be excluded because of disability:

*‘I don't think there's a need to actively make sure more get in, just that it makes no sense to shut them out if they're otherwise willing and smart enough’* [#45]

Three respondents opposed inclusion, on the presumption that someone with a disability or chronic illness would be a less able doctor. Elements of fairness in a highly competitive process were also invoked:

*“There are plenty of able candidates in the application pool”* [#117]

Time, expense and effort involved in educating those who might never practice medicine were also cited:

“*We have to be realistic. We should not build up people's hopes or spend valuable dollars on giving places to people who may never be able to perform the career they have chosen”* [#41]

One person questioned who would benefit from a more inclusive medical profession, implying that a lesser standard of care would be provided:

“*Advantages for whom? Doctors are there for the community. The community should expect only doctors who can function at a very high level*” [#97]

### Life experiences of disability promotes real empathy in doctors

More than three quarters (81%, *n* = 172) agreed that being inclusive of people with disability or chronic illness would be an advantage in practicing medicine. Many of these respondents described people with ‘*real life experiences’* of illness or disability as bringing empathy to the profession. One stated this as *“a unique and currently undervalued perspective to the profession of medicine” [#63].* A common sentiment about the value of lived experience to inform knowledge and practice was shared by others:

*“These people who know more about their own illness rather than just what has been taught, they have firsthand experience enabling better treatment”* [#78]

Empathy would be evident in caring for patients on the basis of better “*understandings of what it means to be unwell” [#16]*, with one emphatically stating:

“*People with disabilities are probably the best people TO study medicine for they have gone through and seen how the hospital system runs and know what it is like to be in a similar position to the patient*” [#94]

Respondents suggested that doctors with lived experiences of being ill or disabled in the community would also be able to make health care experiences ‘*less intimidating*’ because they would appreciate many of *‘the barriers that people face*’ and their ‘*difficulty with access*’.

*“If my doctor told me they had it too, I would personally feel like they understood completely what it's like, not just giving me what they know from a text book”* [#80]

Additionally, some stated that students and doctors with disability may be more aware of these conditions in their peers and colleagues:

“*There is nothing like personal experience to deepen one's understanding and compassion about pain, suffering and health and provide mentorship to others*” [#33]

### Medical career considerations for those with disability

Almost three quarters of respondents (74.7%, *n* = 136 of 182) agreed that a person with a disability or a chronic condition should be encouraged to study medicine, while 39 (21.4%) were unsure and seven (3.8%) said no. Some suggested that ‘becoming a doctor’ would be particularly relevant to individuals with disability or chronic illness. The opportunity would provide meaningful employment for individuals, be enriching and improve their quality of life, as well as provide specialised skills for particular groups. For example, a *‘Deaf doctor who can sign would be of benefit to the Deaf community [90]’.* The diverse employment offered by a medical qualification was highlighted by others:

“*Because medicine encompasses many fields, such as research & development, teaching, ethics, clinical & mentoring fields, even those people who may have poor communication skills, such as autism spectrum disorders or learning disabilities, still may be incredibly creative and brilliant in solving other problems humans encounter & so should therefore not be discounted*” [#18].

There was a common thread throughout that medical students with disability should carefully consider suitable areas of practice. Some considered specific medical specialties being less suitable for people with particular impairments because of the effect on what they perceived as key skills in that specialty*:*

*“Some of these are dependent on what speciality they practice in, someone with severe arthritis may cope as a GP, but not as a surgeon” [#12]*

When asked about being treated by doctors with specific conditions, most were comfortable with a doctor using a wheelchair (92.4%), having a physical disability (89%) or hearing impairment (93.6%), while about half were comfortable with doctors having mental illness such as schizophrenia (50%) or vision impairment (50.6%). Comfort with blindness was related to the need for the practitioner to pick up visual signs:

*“I don't care if my psychologist is blind, I don't want a blind GP” [#7];*

*“My issue would be if they're blind and they need to see something on me that they needed to treat” [#34]*

With mental illness, some suggested that individual ability to perform would need to be considered carefully, recognising the job demands on medical professionals.

### Medical role models to address disabling social barriers

Several respondents held the view that the medical profession could play an important role in addressing disabling social barriers to inclusion by employing doctors with lived experiences of disability and chronic illness, which could benefit the wider community through increased equality. This notion included the ideal that the profession should mirror local populations:

*“The medical profession should reflect the diversity in the community*” [#7]

“*The general population is a wide range of people, and doctors should reflect tha*t” [#29]

Some respondents suggested that addressing barriers to inclusion of people with disability in the medical profession could address disabling barriers more broadly and help to normalise disability in society.

*“May help change attitudes by the very fact that they have real life experience of the condition/s they face every day*” [#62]

It was suggested that medical professionals with disabilities could reduce stigma and stereotyping about disability and mental illness; they could act as advocates for patients with disability as well as being role models and sources of information for colleagues:

“*The potential for this in regard to peer worker understanding of the people they provide services to is a benefit to society*” [#30]

### Responsibility to monitor own health and ability to perform

Some respondents however, were more reserved about inclusion, suggesting that ‘*risk should be measured against the gain*’ and only if a student was *‘entirely capable, then of course they should be encouraged*’ [#71]. Another respondent suggested:

“*This would need to be determined on a one to one basis. The person need to be able to address the inherent criteria of the course and the field of medicine in which they want to work*” [#40].

The academic capacity expected from individuals in an intellectually demanding profession was a concern for some, in terms of risk for patients but also for the student:

“*A person who does not have the intellectual capability to study medicine should not; a lack of intellectual capability would pose a risk to the person and potential future patients*” [#47]

“*If they realistically won't be able to keep up with the intellectual, physical and emotional demands of the profession, encouraging the study of medicine seems almost cruel as you're setting the individual up to fail*” [#71].

Concerns also centred on the significant responsibilities of doctors and expectations to function in often stressful environments. Even if medical students with disabilities were able to pass exams, there was uncertainty about whether they would be able to practice medicine on graduation or have a long career as a doctor.

The safety and wellbeing of medical students undertaking demanding medical training was also raised:

“*Some chronic mental illnesses result in major issues in medical school…”. “Not because they aren't bright or wouldn't be good doctors when they are functioning well, but because of the hugely detrimental effect the course can have on their health and their ability to practice safely*” [#9]

It was acknowledged that some mental health conditions were more complex to manage even with good treatment. One respondent reflected on own experiences, and that there may be periods when doctors needed care and perhaps could be unsafe or unable to care adequately for patients:

*“I say that because as someone who has suffered from depression there are times when I was a danger to myself and wouldn't have been able to provide patients [if I were a doctor] with the care I needed. Same goes for bipolar.” [#44]*

An overarching proviso was that if individuals monitored their own health and undertook treatment when required, there should be no barriers to being a doctor.

*“As long as they can demonstrate ability to understand the work and make decisions under a rational mind. If say they have depression but make sure to take care of themselves when they need to, all good” [#80]*

## Discussion

Overall respondents were positive towards inclusion of those with disability as medical students and doctors. We note that the sample was skewed towards a highly educated population whose views might be more reflective of, and informed by, equal opportunity policies as well as being more trusting of higher education. However, our analysis of the qualitative data also shows that while respondents believed that people with disability have much to offer the medical profession, they believed that standards should be upheld. Suggestions included that only individuals who are assessed as able to manage the demands of studying and engaging in clinical practice should be encouraged to study and practice medicine. In our previous research we have noted though, that evaluation of such capacity to align with standards typically focuses on the individual, and “neglect the wider social and cultural factors that contribute to the disabling system” [6].

Findings also suggest that people with disability or chronic illness may have unique abilities and experiences to contribute to medical practice, and that a diverse medical profession would better relate to the community it serves. These findings help to address a lacuna in research on students and doctors with disability, and while this research was based on the Australian community, findings reflect the arguments for inclusion made in the UK and US in the past decade for example [2, 4, 21, 22]. Respondents also highlighted that there was a balance to strike between the risks and gains of inclusion. Concerns were raised by some respondents that particular impairments could be problematic given the demands of medical practice, and that some individuals would require substantial support and even monitoring of their health and capacity. There was an expectation that the responsibility for achieving balance rested with medical schools, by assessing each applicant on a case by case basis in the selection process and in monitoring student capacity until graduation. In Australia there are currently no clear national guidelines for such processes in program delivery, and it is likely that practices in individual schools differ. Our finding that the public rely on Australian medical schools to select and monitor for the safety and competency of all medical students is reflected in Australian accreditation standards [23]. However, to uphold accreditations standards, we suggest that an approach should be adopted that circumvent perpetual “resistance to inclusion of medical students with disabilities…” [6]. Evaluations of students should be made with understanding of individual context, and with considerations for reasonable accommodations as required of universities and medical schools in many countries.

The Association of American Medical College’s [22] stringently argues that schools should develop policies and procedures for better including students with disability that apply across the educational continuum from admissions, to learning the basic sciences, to clinical rotations. In considering the lack of change in the composition of medical student and graduate cohorts despite the inclusive missions of many medical schools, we suggest that explicit policies for supporting and reporting retention and graduation outcomes, directed by accreditation standards, might help a greater diversity of medical students to manage the requirements of medical curricula.

The selection of students suitable for medicine, and the responsibility for monitoring performance of all students is discussed in UK literature in relation to the obligation of medical education and training bodies to be accountable for producing competent graduates [4]. Boursicot et al. [4] maintain that the ‘competency argument’ is “one of the biggest challenges to skill-based professions such as medicine” in terms of the inclusion of people with disability, because it upholds that “disabled people will be unable to deliver adequate levels of healthcare” (p. 22). This is an argument also voiced by respondents in this study who opposed inclusion. Widening participation policies challenge the previously “closed and elitist nature of medicine” [4, p., 19], but also raises the challenge of how healthcare can be delivered more collaboratively and coordinated across specialities with a more diversely abled profession [see Albrecht, 2]. In an era of interprofessional and team-based care, inclusion of health care professionals of differing abilties should not only be possible but potentially lead to better quality care. Further work will be needed to address barriers such as stigma towards disability and assumptions about traditional one practitioner-one patient models of healthcare delivery that persist even in well-educated community members, and to develop more inclusive educational practices in health professional education.

Our data suggest that community members believe that doctors living with illness or disability will provide more holistic and empathic care, which provides supporting evidence for arguments made by other authors [1, 5], highlighting a desire for a distinctive patient centred approach to healthcare [2]. Medical students and trainees with disabilities can help doctors and fellow students to better understand diversity and real life experiences of illness and diagnosis [7]. This understanding was recently illustrated by a young Australian doctor with quadriplegia, who graduated from medicine after sustaining a spinal cord injury as a medical student [24]. His story describes the learning curve for academics, clinicians and other medical students, and how the perceived risks of providing novel adjustments were measured against the gains of including and training a highly motivated and otherwise able ‘disabled’ student.

The importance of a medical profession that values diversity was emphasised in our findings; a point that has been argued long and keenly, for example by Mercer and Pinder [25], who suggested that the medical profession should progress from preserving doctors as the ‘perfect being in the white-coat’. They argue that personal experience of successfully managing with a disability places doctors in a uniquely useful position, and that such experience should be viewed as a resource. Others have suggested that an increase in the number of doctors with disabilities would perhaps help health professional colleagues improve their consideration for and engagement with disabled people generally [25], setting a precedent for a more inclusive society more broadly [26–29].

### Limitations

This study had a limited number of participants, who as described, were mostly female, highly educated and in professional occupations. Most resided in the Greater Western Sydney area, but differed from this population, of which 44% reportedly have no formal qualification, and only 20% have an undergraduate degree or higher [19]. Repeating the survey with a larger, stratified sample might represent the Australian population more accurately. However, for medical school planning, it is important that the mission of the school reflects the views of the communities which it serves. Our findings could, for example, specifically inform the policies and practices of medical schools in the Greater Western Sydney. The sample nevertheless reflects those who are more likely to respond to surveys, and are invested in medical education. Where expressed, negative views and concerns have been included in the findings to illustrate the range of responses.

Most respondents had personal experiences with disability, through occupation or people close to them. Findings from standardised scales [27–29] show that having experience with disability or knowing someone with disability is associated with more positive attitude towards disability. However, this survey may well be representative of the Australian population; almost 20% of Australians [4.2 million people] report a disability [11], so most people in Australia can be expected to know at least one person with disability.

## Conclusion

Our findings suggest community support for inclusion of people with disability or chronic illness in medical education and practice, with a key reason being that their life experiences offer true understanding and empathy with patients. These findings provide evidence for arguments for inclusion from UK and US. In Australia, the number of students with disability medical education is incongruent with other higher education courses in Australia, with little effect from widening participation policies. The reasons are yet to be understood, but our findings suggest that there is strong support for medical schools to develop and implement policies to better and more actively include students with disability, and to support all students to achieve their potential and provide value as medical practitioners, but also to address concerns about how some students with disability or chronic illness may manage the demands of study and professional practice. The public expectation that medical schools carry the responsibility for selecting capable students and monitoring their health, well-being and performance applies to all students, but also calls for Australian medical schools to review their inclusive practices, and to develop polices and guidelines similar to those implemented in the UK [7] and the US [21] for admitting and supporting students with disability through to graduation and beyond.

## Additional file

Additional file 1: Survey questions. (DOCX 48 kb)

## Data Availability

The datasets collected and analysed for this paper can be made available from the corresponding author on reasonable request.

## Abbreviations

GP: General practitioner
UK: United Kingdom
US: United States
