# Supplementary material for: “A doctor who really knows …”: a survey of community perspectives on medical students and practitioners with disability
Source: BMC Med Educ. 2019 Jul 29;19:288. doi: 10.1186/s12909-019-1715-7 (PMC6664718; doi:10.1186/s12909-019-1715-7)
Supplement: Supplementary file 1 — Survey questions. (DOCX 48 kb) [file 12909_2019_1715_MOESM1_ESM.docx]

**Additional file 1: Survey questions**

**Knowledge and views on disability in the medical profession**

** Adapted from Park et al 2007 [1]*

*** Adapted from Roberts et al 2004 [2]*

**** New*

**Question 1***

People have different ideas about what it means to be disabled. Which of the following persons would you consider to be disabled? (Table with 15 disabling conditions. Answer option yes/no/not sure)

**Question 2***

Do you know anyone with a long standing physical, mental health condition or disability?

(Long standing means: has lasted at least 12 months or is likely to last at least 12 month)

**Yes □ No □ If no, go to question 6**

**Question 3*****

Is this person, a person with: (Tick all that apply in table with 15 disabling conditions and ‘other’ option)

**Question 4*****

Is this person: (tick all that apply: your partner, child, close relative, close friend, colleague/co-worker, boss, health service provider, person in local community, someone else you know well)

**Question 5*****

Have you ever felt that this person was discriminated against or treated poorly on the basis of this condition or disability? **Yes □ No □**

Optional – if yes, provide a brief example: **____________________________________________**

**Question 6***

Generally speaking, how much prejudice do you think there is in [Australia] against people with the conditions listed in the table below? (please tick): (table with 15 disabling conditions, options A lot, a little, hardly any, none, not sure)

**Question 7*****

Generally speaking, do you think that a person with a disability or chronic condition should be able and encouraged to pursue an education towards a career of their choice? **Yes □ No □**

**Question 8****

Do you think that a person with any one of the following conditions could enrol and successfully complete a degree in medicine? (Table with 15 disabling conditions, options yes, no, not sure)

Optional – briefly explain your response(s): ________________________________________

**Question 9***

How would you feel a person with one of the conditions listed below was your doctor? (table with 15 conditions options, very comfortable, fairly comfortable, fairly uncomfortable, very uncomfortable, not sure)

Optional – if you selected uncomfortable, briefly list some reasons: ________________

**Question 10****

To your knowledge, have you ever been seen or treated by a doctor who had any of the conditions listed below? (same 15 conditions + other, yes or no)

**Question 11***

Do you think a person with any one of the following conditions could successfully practice as a doctor? (Table with same 15 conditions, answer options yes, no, not sure)

Optional – briefly explain your response(s): **______________________________**

**Question 12*****

Generally speaking, do you think a person with a disability or a chronic condition should be encouraged to study medicine, if they are academically capable? **Yes □ No □**

Optional - comment on your answer: **__________________**

**__________________________________________________________________________________**

**Demographic information:**

- What is your age?
- Are you: Male □ Female □
- What is your Nationality/Ethnicity? ____________________________________________________
- What is your highest level of education? Primary □ Secondary □ Certificate □ Trade certificate □ Diploma □ Graduate degree □ Post-graduate degree □
- What is your occupation and current position? ____________________________________________
- What is your postcode: _________
- Do you consider yourself to have a long standing physical condition, mental health condition or disability? (Long standing means: has lasted at least 12 months or is likely to last at least 12 month)

Yes □ No □

1. Park A, Curtice J, Thomson K, Phillips M, Johnson M. Disabling attitudes? Public perspectives on disabled people. In: Park A, Curtice J, Thomson K, Phillips M, Johnson M, editors. British Social Attitudes: The 23rd Report. London: SAGE Publications Ltd; 2007. doi: <http://dx.doi.org/10.4135/9781849208680.n8>
2. Roberts T, Butler A, Boursicot K. Disabled Students, Disabled Doctors-Time for a Change?: A Study of Different Societal Views of Disabled People's Inclusion to the Study and Practice of Medicine. Higher Education Academy: Medicine, Dentistry and Veterinary Medicine; 2004. <http://www.academia.edu/208774/Disabled_students-_disabled_doctors_time_for_a_change_A_report_on_a_study_looking_at_societal_views_on_the_admission_of_disabled_people_to_medicine_2004> Accessed January 15 2016
